# Luminal MCF-12A & myoepithelial-like Hs 578Bst cells form bilayered acini similar to human breast

**DOI:** 10.4155/fsoa-2018-0010

**Published:** 2018-06-28

**Authors:** Anne Weber-Ouellette, Mélanie Busby, Isabelle Plante

**Affiliations:** 1 INRS – Institut Armand-Frappier, Laval, Quebec, H7V 1B7, Canada

**Keywords:** 3D co-culture model, acini, acinus, breast, epithelium, mammary gland

## Abstract

The epithelium's functional unit is the bilayered acinus, made of a layer of luminal cells, surrounded by a layer of basal cells mainly composed of myoepithelial cells.

**Aim:**

The aim of this study was to develop a reproducible and manipulable 3D co-culture model of the bilayered acinus *in vitro* to study the interactions between the two layers.

**Materials & methods:**

Two different combinations of cell lines were co-cultured in Matrigel: SCp2 and SCg6 mice cells, or MCF-12A and Hs 578Bst human cell lines.

**Results:**

Confocal microscopy analysis showed that only MCF-12A and Hs 578Bst cells could form some bilayered acini. This *in vitro* bilayered acini model will allow us to understand the role of interactions between luminal and myoepithelial cells in the normal breast development.

## Lay abstract

The breast epithelium, which is the structure that produces the milk during lactation, is composed of two layers of cells: an inner layer of luminal cells, surrounded by an outer layer of basal cells mainly composed of myoepithelial cells. Studies have demonstrated that the bidirectional crosstalk between these two layers of cells is crucial for the proper development and function of the breast. This paper presents a new innovative *in vitro* model that recapitulates the structure of the breast epithelium to further understand the interactions between luminal and myoepithelial cells, and how their dysregulation relates to developmental defect and breast cancer.

## Graphical abstract

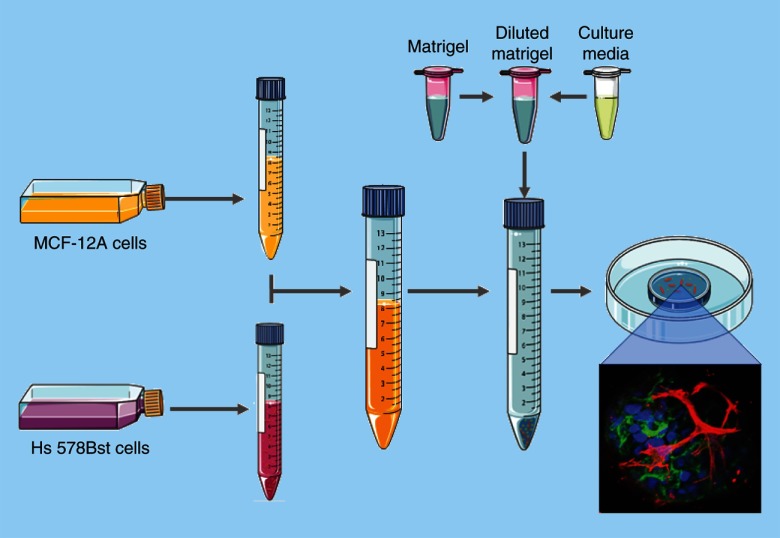

The human mammary gland consists of two compartments: the stroma and the epithelium. The mammary epithelium is organized in a ramified lobulo-alveolar system. In humans, 15–20 lobes in each breast converge in ducts toward the nipple. Each lobe is itself subdivided in lobules, and each lobule consists of many acini grouped together [1]. The acinus is considered to be the functional unit of the mammary gland. The whole ramified lobulo-alveolar system of the epithelium consists of an inner layer of luminal cells bordering a central lumen and surrounded by an outer layer, mainly comprised of myoepithelial cells. In the acini, the myoepithelial cells form a basket-like network surrounding the luminal cells [2]. For many years, it was thought that the role of myoepithelial cells was limited to milk transport upon contraction during lactation; we now know that they are required for the proper development and function of the mammary gland [3–5]. As a result, dysregulation of myoepithelial cells functions has been associated with the loss of a polarized epithelium, developmental defects and tumorigenesis [6–10]. They are thereby considered as ‘natural tumor suppressors’ [11,12]. All these functions are critically dependent on bidirectional communication between myoepithelial and luminal cells [3–5,13]. However, few models allow for the study of the mechanisms involved in these heterocellular interactions.

The epithelium is separated from the surrounding stroma by a basement membrane [14]. The basement membrane is a type of specialized extracellular matrix (ECM) mainly composed of type IV collagen and laminin-1 [15]. The stroma is comprised of ECM, of mesenchymal and immune cells, as well as of blood and lymphatic vessel cells [16]. In addition to physically supporting the epithelium, the stroma's components directly transmit signaling cues to the epithelium including to the cytoskeleton of the cell through transmembrane receptors such as integrins, which ultimately impinges on chromatin and nuclear function to maintain tissue integrity [17]. Elements from the stroma influence the development of the epithelium through physical but also chemical interactions [18]. Hormones and growth factors influence mammary gland development and function by signaling through receptors present exclusively in the stroma, only in the epithelium or in both compartments [19–23]. Moreover, the stroma directly surrounding the epithelium, namely, the basement membrane and ECM, guides the development of the epithelium, and the epithelium reciprocally influences its microenvironment [24]. Levels of collagen-I, collagen-IV and laminin-5 vary sequentially during ductal and alveoli development, and each estrus-associated proliferation-regression cycle is accompanied by basement membrane remodeling [25], suggesting an important role of the microenvironment in organogenesis. In the breast, the stroma ECM is thus considered as an active participant, rather than a passive bystander, in cellular differentiation. Hence, the environment in which cells grow, or in which cells are cultured *in vitro*, impacts their morphological organization and tissue-specific functions [16]. However, traditionally, cultured cells are grown in a 2D monolayer and not in ECM. They adhere and grow on a flat surface, and adopt a flatter and more stretched out phenotype compared with *in vivo*. This abnormal cell morphology alters cellular processes such as cell proliferation, differentiation, and apoptosis, as well as gene and protein expression [26]. Consequently, cells cultured in 2D may not behave as they would in tissues [27].

To overcome the limitations of traditional 2D cell culture and to adequately mimic the *in vivo* microenvironment, cells can be cultured in 3D. This type of culture allows cells to freely assemble in multidimensional structures, called spheroids, using a scaffold/matrix or in a scaffold-free manner [28]. By adopting this *in vivo*-like 3D relationship to each other, cells can better form cell–cell and cell–ECM interactions, establish appropriate cell-signaling pathways to maintain tissue function and mimic the cellular processes occurring in the human body [28]. This difference between 2D and 3D culture regarding cellular processes and response, through the modulation of gene expression, has been observed several times [29–31]. In fact, distinct patterns in gene expression profiles between tissue samples and cell lines of varying phenotypes demonstrated adaptation of cells to their culture microenvironment [32,33].

Most 3D cell culture systems mimicking mammary gland acini are monocultures, using only the luminal cells [34–36]. Yet, to thoroughly recapitulate the histological complexity of the normal human mammary gland, luminal cells must crosstalk with EMC or scaffolds, but also with myoepithelial cells through physical interactions and by paracrine signaling [37]. These interactions are critical for the proper polarization of the luminal cells [10]. Heterotypic models of the human mammary gland have been developed using luminal MCF-10A cells, adipocytes and human fibroblasts in a mixture of laminin-rich basement membrane extract (lrBME) matrix/collagen on porous silk protein scaffold [38]. While this model represents progress toward an *in vitro* acinus-like structure composed of multiple cell types, it uses complex matrices and scaffolds, whereby myoepithelial cells are absent. Bilayered acini composed of a mix of purified human luminal and myoepithelial cells isolated from normal mammary glands have also been developed, with or without fibroblasts [10,39]. However, because they are formed using primary cultures, these models have some limitations. For instance, they are hardly genetically manipulable, access to human tissues is difficult, and there is a great patient-to-patient variability. There is thus still a great need for a simplified, optimized, genetically manipulable, reproducible and physiologically relevant model to recapitulate the normal structure of the functional unit of the human mammary gland – the bilayered acinus [40]. By using commercially available cell lines instead of primary cells, such a model will be accessible to the scientific community and allow more mechanistic studies in the understanding of the biology of the human bilayered acini.

This study aimed to develop a model representing the breast bilayered acini that can be genetically manipulated and easily reproduced by using cell lines. Here, two combinations of nontumorigenic cell lines were investigated: the human luminal and myoepithelial-like cells MCF-12A and Hs 578Bst; and the murine luminal and myoepithelial cells SCp2 and SCg6 [41].

## Materials & methods

### Cell lines

MCF-12A cells (ATCC^®^ CRL-10782) and Hs 578Bst cells (ATCC HTB-125) were purchased at ATCC (ATCC, VA, USA). SCp2 and SCg6 cells were a gift from Calvin Roskelley (University of British Columbia [UBC]). MCF-12A cells were maintained in phenol red-free Dulbecco's modified Eagle's medium Ham's F12 (DMEM/F12) culture medium (21041025, ThermoFisher Scientific, IL, USA) supplemented with 5% (v/v) horse serum (ThermoFisher Scientific, 16050–122), hEGF recombinant (20 ng/ml) (PHG0311, Invitrogen, MA, USA), hydrocortisone (500 ng/ml; H0888, Sigma-Aldrich, Oakville, Ontario, Canada), insulin (10 μg/ml; Sigma-Aldrich, C8052), cholera toxin (100 ng/ml; Invitrogen, 12585014) and propagated according to ATCC guidelines. Hs 578Bst cells were maintained in Hybri-Care medium (ATCC 46-X™) supplemented with 10% (v/v) activated fetal bovine serum (098150, Wisent Bioproducts, Saint-Jean-Baptiste, Quebec, Canada) and mouse EGF (EGF from murine submaxillary gland, 30 ng/ml; Sigma-Aldrich, E4127) and propagated according to ATCC guidelines. SCp2 and SCg6 cells were grown in DMEM/F12 medium (Sigma-Aldrich, D2906) supplemented with insulin (5 μg/ml) and fetal bovine serum (5% v/v).

### Western blot analysis

Cell monolayers were washed twice with phosphate buffered saline (PBS) before the addition of lysis buffer (Tris 50 mM, NaCl 150 mM, 0.02% sodium azide, 0.1% sodium dodecyl sulfate (SDS), 1% Nonidet P40, 0.5% sodium deoxycholate, pH 8) supplemented with NaF 1.25 M, NaVO3 1 M and Halt Protease and phosphatase cocktail inhibitor (Fisher Scientific, Ontario, Canada). Cells were scraped, collected and incubated on ice for 5 min. Cell lysates were centrifuged for 10 min at 2500 r.p.m. at 4°C. The supernatants were aliquoted and stored at -80°C until further processing. Lysate protein concentrations were measured using a bicinchoninic acid protein assay reagent kit (Thermo Scientific #23227). Protein samples were resolved on stain-free acrylamide gels (TGX Stain-Free FastCast Acrylamide kit, 10%, Bio-Rad, Ontario, Canada) and transferred onto polyvinylidene fluoride membranes (PVDF). Membranes were blocked with Tris Buffered Saline (TBS)-Tween 20 (0.1%; Fisher Scientific) containing 3% bovine serum albumin (BSA) or dry milk, according to manufacturer instructions, for 1 h and incubated overnight at 4°C with the following primary antibodies: anti-E-cadherin (#3195; Cell Signaling Technology, MA, USA), anti-Cytokeratin 18 (K18) (#ab52948; Abcam, MA, USA), anti-Cytokeratin 14 (K14) (#MS-115-P1ABX; Thermo Scientific, Cheshire, UK), anti-alpha-Smooth muscle actin (α-SMA) (#M0851; Dako, Glostrup, Denmark), anti-calponin-1 (#17819; Cell Signaling Technology), anti-caldesmon-1 (#12503; Cell Signaling Technology) and anti-p63 antibody (#13109; Cell Signaling Technology). Bound primary antibody was detected using HRP-conjugated secondary antibodies (goat-anti-rabbit [#7074] or horse-anti-mouse (#7076); Cell Signaling Technology) followed by visualization and quantification using a Bio-Rad ChemiDoc MP System (Bio-Rad Laboratories, Ontario, Canada). Chemiluminescent signals were detected using Clarity western ECL substrate (Bio-Rad Laboratories) and analyzed using Image Lab software (Bio-Rad Laboratories).

### 3D-embedded cell cultures

For 3D cultures, cells were embedded in solubilized basement membrane extract – Matrigel (Corning^®^ Growth Factor Reduced Basement Membrane Matrix from Engelbreth-Holm-Swarm mouse sarcoma; *CB40230C*, Corning, NY, USA) at a cell density of 75,000 cells/100 μl of Matrigel. When required, Matrigel was diluted to different final concentrations by adding ice cold growth medium. Experiments were carried out in 35 mm glass bottom poly-_D_-lysine-coated dishes, 14 mm microwell (P35GC-0–10-C, MatTEK Corporation, MA, USA). Plates were manually evenly precoated on ice with 10 μl of Matrigel using the tip of a pipette. Dishes were left in an incubator at 37°C to allow the Matrigel to congeal for 30 min. For co-culture experiments, cell suspensions from both cell types were counted and the proper number of each cell type mixed in the same tube. Cell suspension containing both cell types were centrifuged at 125 g at room temperature for 7 min, the supernatant was removed and the tube was gently flicked to detach cells from the bottom of the centrifuge tube. The Matrigel was added directly to the cell pellet, gently mixed and the Matrigel-cells suspension was distributed rapidly in the microwells precoated with Matrigel. Dishes were incubated for another 30 min at 37°C to allow Matrigel to congeal, then the culture medium was added to cover the entire polymerized Matrigel/cell mix. The culture medium was changed every 2–3 days for 14 days.

### Immunolabeling of 3D-embedded cell cultures

Culture medium was aspirated, and embedded cultures were rinsed twice with PBS. Cells were fixed in formaldehyde 4% for 10 min and permeabilized in PBS-Triton X-100 (0.5%) for 60 min. After being rinsed twice (10 min each) with PBS-glycine (0.1%), cells were incubated in blocking solution (2% BSA dissolved in PBS with 0.1% of Tween 20 and 0.1% of Triton X-100). Primary antibodies were diluted in blocking solution and cells were incubated with primary antibody for 120 min at room temperature or overnight at 4°C (E-cadherin [#3195s] 1/200 [Cell Signaling, MA, USA]; alpha-smooth-muscle-actin (α-SMA) mouse mAb [M0851] 1/300 [Agilent, CA, USA]). Immunolabeling was followed by four washes with washing solution (PBS containing Tween20 [0.1%], BSA [0.1%] and Triton X-100 [0.5%]), for 5 min each. Cells were incubated with the appropriate secondary antibodies for 60 min (anti-rabbit IgG Alexa Fluor 488 [#4412s], anti-mouse IgG Alexa Fluor 555 [#4409s] both used at 1/1000 [Cell Signaling], or anti-rabbit IgG DyLight 488 [#35552] used at 1/200 [ThermoFisher Scientific]). Secondary antibody labeling was washed three-times with washing solution and one time with PBS, for 5 min each. Nuclei were stained with 4′, 6-diamidino-2-phenylindole in PBS. Embedded cultures were mounted with Fluoromount-G (0100–01, SouthernBiotech, AL, USA). Fluoromount-G was added in enough volume to cover the embedded cultures and fill up the microwell. Coverslips were added and mounted cultures were placed horizontally overnight at 4°C for 8 h in the dark. Coverslips were sealed 24 h after mounting with hot glue. Immunofluorescence images were obtained with a Nikon A1R+ confocal microscope (Nikon Canada Inc, Mississauga, Ontario, Canada) and analyzed using NIS-elements software (Nikon Canada Inc).

### Cryosections from embedded 3D cultures

Culture medium was removed and embedded cultures were washed twice with PBS, prewarmed at 37°C. The matrix was detached from the microwell with a spatula, quickly placed on top of a layer of Tissue Freezing Medium (3801481, Leica Biosystems, Wetzlar, Germany) in a cryomold, and then covered with a second layer of Tissue Freezing Medium to fill the cryomold. The resulting block of cryomatrix containing the cells embedded in the matrix was placed at -80°C until use.

### Masson's trichrome staining of cryosections

Embedded acini cryosections (8 μm) were fixed in Bouin's solution overnight and then rinsed with water for 5 min. Sections were stained sequentially with Weigert's iron hematoxylin for 10 min, Biebrich scarlet-acid fuchsin for 15 min, phosphomolybdic-phosphotungstic acid for 20 min and aniline blue (5 min), and washed with water between all coloration steps. Finally, the sections were treated with 1% acetic acid for 5 min, dehydrated in an alcohol series, cleared in xylene for 5 min and mounted using Permount (SP15100, Fisher Scientific, Ontario, Canada). Slides were dried afterwards for a minimum of 4 h.

### Immunolabeling of cryosections

Embedded acini cryosections (8 μm) were fixed in formaldehyde 4% for 15 min and blocked in 3% BSA dissolved in TBS-Tween20 (0.1%). Primary antibodies were diluted in TBS-Tween 0.1%, and sections were incubated with primary antibody for 60 min at room temperature. Sections were incubated with β-catenin (L54E2) mouse mAb (#2677s) 1/200 (Cell Signaling). Immunolabeling was followed by three washes with TBS-Tween 0.1%. Sections were incubated with the secondary antibody (anti-mouse IgG Fab2 Alexa Fluor 647 [#4410s] used at 1/1000 [Cell Signaling]). Nuclei were stained with 4′,6-diamidino-2-phenylindole in TBS-Tween 0.1%, and slides were mounted with Fluoromount-G (SouthernBiotech, 0100–01). Slides were placed at 4°C for 8 h in the dark. Immunofluorescence images were obtained with a Nikon A1R+ confocal microscope (Nikon Canada Inc) and analyzed using NIS-elements software (Nikon Canada Inc).

## Results

### Human MCF-12A & Hs 578Bst cells showed more differentiated phenotypes than mice SCp2 & SCg6

We first confirmed that the selected cell lines were representative of luminal and myoepithelial cells by evaluating the expression of various differentiation markers (Supplementary Figure 1). Results showed that in human cells, E-cadherin and K18 were present only in luminal MCF-12A, whereas in murine cells, E-cadherin was present in both SCp2 and SCg6 (Supplementary Figure 1A & B). Calponin-1 and α-smooth muscle actin (α-SMA) were expressed only in myoepithelial cells, as expected, while K14 was present in both SCp2 and SCg6, but was not detected in Hs 578Bst cells (Supplementary Figure 1A, E & F). p63, a marker specific to myoepithelial cells, was expressed in Hs 578Bst but not in SCg6 cells (Supplementary Figure 1C). In addition, the muscle-specific form of caldesmon-1 was detected in Hs 578Bst, while low levels were also observed in both SCp2 and SCg6 cells (Supplementary Figure 1D). Together, these results demonstrated that while MCF-12A and Hs 578Bst cells expressed markers typical for luminal and myoepithelial cell types, respectively, some markers were present in both SCp2 and SCg6 cells (Supplementary Figure 1).

### MCF-12A or SCp2 luminal cells embedded in Matrigel form spheroid structures

**Figure 1.**
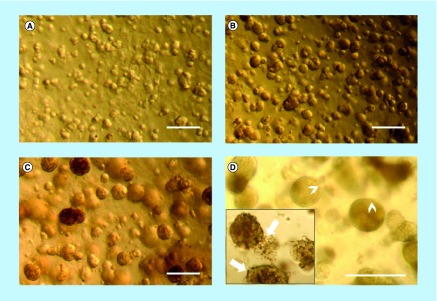
**MCF-12A cell embedded in Matrigel gradually form acini-like structures.** Optical microscopy images of MCF-12A. **(A)** Cells appeared in small clusters after 24 h. They gradually formed acini-like spheroids after 4 days **(B)**, which grow in size after 10 days **(C)**. Fourteen days after being embedded in Matrigel, spheroids preserved their size and some of them were more defined, with clear edges (**D;** arrowheads), while others were less defined (**D;** insert, arrows). Scalebars: 250 μm.

We then wanted to confirm that luminal MCF-12A and SCp2 cell can form acini-like structures, as reported in the literature [13,35]. To do so, MCF-12A were embedded in a 3D matrix consisting of a mix of cell culture medium and basement membrane extract, commercially known as Matrigel. After 24 h in culture, cells already formed small clusters of cells (Figure 1A). After 4 days, small and rounded spheroid structures could be observed (Figure 1B). After 10 days, the spheroids were much bigger in size, and the cells that constituted each spheroid could be distinguished (Figure 1C). After 14 days in culture, spheroids maintained their size, but while some MCF-12A spheroids showed a defined spherical structure similar to the acini of the human mammary gland, others were less defined and characterized by a certain looseness in the structure (Figure 1D). These spheroids also displayed what looks like cellular degradation at their edges (Figure 1D). Likewise, SCp2 cells could form acini-like structures with a lumen (Supplementary Figure 2A & B).

### MCF-12A cells maintained in Hybri-Care culture medium conserved an acini-like structure

**Figure 2.**
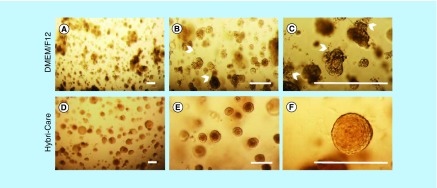
**Hybri-Care culture medium promotes the formation of more defined acini-like structure than Dulbecco's modified Eagle medium/F12 culture medium.** Optical microscopy images of MCF-12A cell embedded in Matrigel after 14 days of culture. When using Dulbecco's modified Eagle medium/F12 medium to maintain cultures, resulting multicellular structures seem smaller, less defined and looser **(A–C**, arrowheads**)** than cells maintained in Hybri-Care medium **(D–F)**. In Hybri-Care medium, acini are bigger, rounder, more defined and more compact. Scalebars: 250 μm.

Because Hs 578Bst and MCF-12A cells are typically grown in different media, and because Hs 578Bst cells are harder to maintain than MCF-12A cells, we wanted to ensure that MCF-12A could still form acini-like structures in Hs 578Bst cells culture media. We thus compared spheroids formed by MCF-12A cells embedded in Matrigel and cultured with either Hybri-Care medium (ATCC^®^ 46-X^™^) or phenol red-free DMEM/F12 medium, the media typically used for Hs 578Bst and MCF-12A cells, respectively. MCF-12A spheroids with defined spherical structure, as well as some with less defined structures, were observed when cultured in DMEM/F12 medium, as previously demonstrated (Figures 1 & 2A–C). Interestingly, when Hybri-Care medium was used to dilute the Matrigel and to culture embedded MCF-12A cells, the cells formed bigger, rounder and more defined acini-like spheroids. These structures were more compact and there was no visual evidence of cellular degradation (Figure 2D–F). These observations suggested that MCF-12A cells can form acini-like structures even more efficiently in Hybri-Care medium. Because SCp2 and SCg6 cells are cultured in the same medium, these optimizations were not necessary.

**Figure 3.**
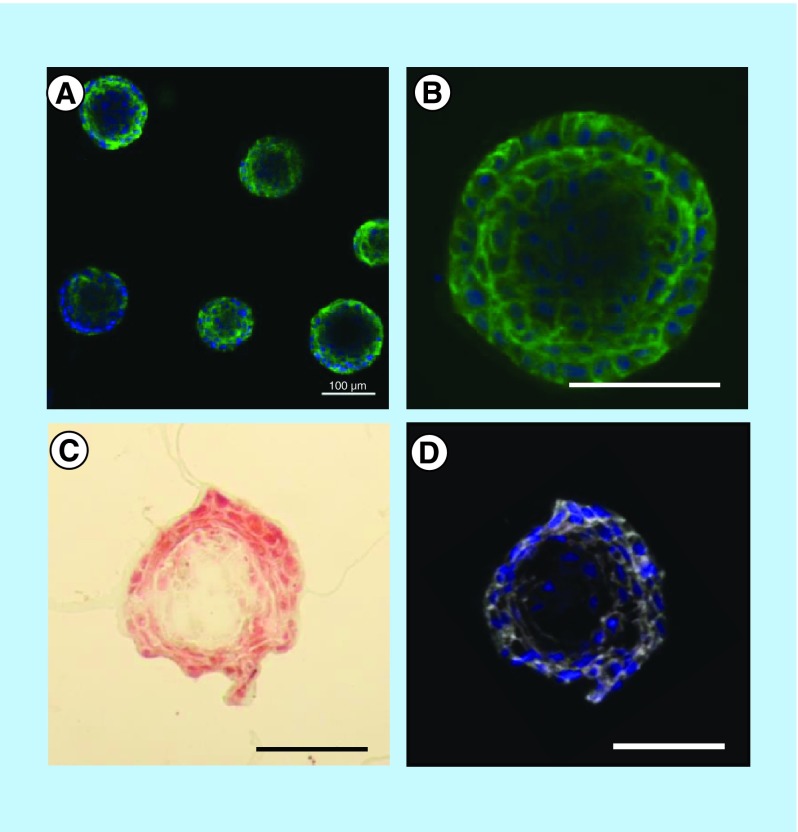
**Presence of a lumen-like cavity in the center of cryosectioned MCF-12A acini.** Confocal microscopy images of acini immunolabeled with an E-cadherin (adherens junctions)-specific antibody (green). Nuclei are stained with 4′,6-diamidino-2-phenylindole (blue). **(C)** Optical microscopy image of acini cryosection stained with Masson's trichrome staining. **(D)** Confocal microscopy image of an acini cryosection immunolabeled with a β-catenin (adherens junctions)-specific antibody (white). Nuclei are stained with 4′,6-diamidino-2-phenylindole. Scalebars: 100 μm.

To ensure that these spheroids maintained the lumen typical of acini, they were analyzed by confocal microscopy. The acini presented lower cell density at their center, suggesting the gradual apoptotic clearance of cells creating a lumen-like cavity (Figure 3). These results were confirmed by cryosections of MCF-12A acini, either stained using Masson's trichrome coloration (Figure 3C) or immunolabeled with β-catenin-specific antibody (Figure 3D). Adherens junctions were formed between luminal cells as demonstrated by the expression of both E-cadherin and β-catenin (Figure 3A, B & D).

### Dilution of Matrigel to different concentrations impacts spheroid structure & immunolabeling

**Figure 4.**
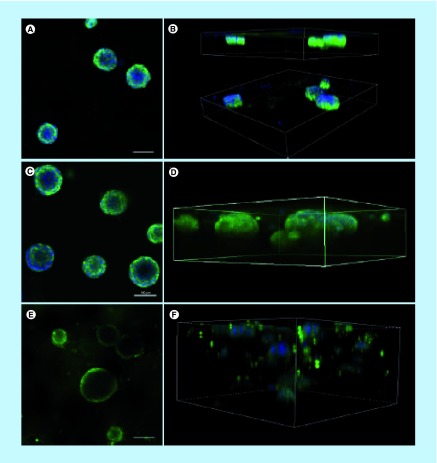
**Matrigel at a concentration of 75% is optimal for 3D culture of MCF-12A cells.** Representative single confocal images **(A, C & E)** or serial Z-stacks **(B, D & F)** of MCF-12A cells embedded in 50% **(A & B)**, 75% **(C & D)** or 100% **(E & F)** Matrigel and immunolabeled with an E-cadherin antibody (green). Nuclei are stained with 4′,6-diamidino-2-phenylindole (blue). Scalebars: 100 μm.

To optimize the immunolabeling of the acini-like spheroids, without compromising their 3D structure, we then defined which Matrigel concentration is most favorable to support round acini formation and to facilitate antibody penetration in the matrix for immunofluorescence imaging. MCF-12A cells were embedded in Matrigel diluted with Hybri-Care medium to achieve concentrations of 50, 75 and 100% of Matrigel (Figure 4). In wells containing 50% Matrigel, the matrix almost completely liquefied during the immunolabeling procedure, resulting in the loss of many acini during the staining procedure. The remaining acini fell to the bottom of the microwell, forming flat structures difficult to properly label with antibodies (Figure 4A & B). On the opposite, in wells containing 100% of Matrigel, the matrix remained rigid in its consistency, resulting in unspecific labeling and smaller acini (Figure 4E & F). Finally, in wells containing a concentration of 75% of Matrigel, the matrix had a soft consistency, but preserved its rigidity through washes, and less nonspecific immunolabeling was observed (Figure 4C & D).

### MCF-12A & Hs 578Bst self-organized in spheroids resembling the bilayered acini

**Figure 5.**
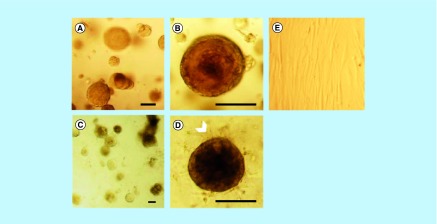
**MCF-12A cells co-cultured with Hs578Bst cells produce acini-like structures when embedded in Matrigel.** **(A & B)** Representative optical microscopy images of MCF-12A cells embedded in Matrigel. **(C & D)** Optical microscopy images of MCF-12A cells co-cultured with Hs 578Bst cells showing acini-like structures. A second layer of cells seems to be surrounding the first layer in some of the acini (arrowhead), suggesting that myoepithelial cells formed bilayered acini with the luminal cells. **(E)** Optical microscopy image of Hs 578Bst cells embedded in Matrigel. Scalebars: 100 μm.

Once culture conditions were optimized for MCF-12A, we co-cultured luminal MCF-12A cells with myoepithelial-like Hs 578Bst cells or SCp2 cells with SCg6 cells. Similar to when they were placed alone in 3D monoculture (Figure 5A & B), MCF-21A formed acini-like structures when simultaneously embedded with Hs 578Bst cells in Matrigel (Figure 5C & D). In the co-culture, however, a second layer of cells seemed to surround some of the acini, suggesting that bilayered acini were formed with both types of cells (Figure 5D, arrowhead). Hs 578Bst cells, when cultured alone, were not able to form spheroids (Figure 5E). On the opposite, SCg6 cells could form spheroids alone or in co-culture with SCp2 (Supplementary Figure 2C–E).

**Figure 6.**
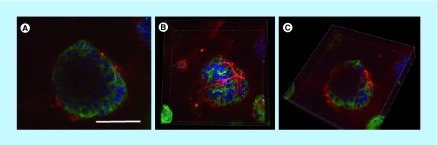
**MCF-12A and Hs 578Bst co-cultured cells form bilayered acini in Matrigel.** **(A–C)** Confocal microscopy images of MCF-12A and Hs 578Bst cells forming a 3D bilayered acini when co-cultured in Matrigel. Acini were immunolabeled with an E-cadherin-specific antibody (green) and an smooth muscle actin-specific antibody (red). Nuclei are stained with 4′,6-diamidino-2-phenylindole (blue). Representative single confocal image **(A)**, serial Z-stack **(B)** and a truncated view of a Z-strac **(C)**. The myoepithelial-like cells Hs 578Bst have a stellate phenotype, forming a discontinuous basket-like network around the MCF-12A luminal cells. The truncated view suggests the presence of a lumen in the center of the bilayered acini. Scalebar: 100 μm.

To confirm that these acini were indeed bilayered, we performed immunolabeling using distinct epithelial (E-cadherin) and myoepithelial (α-SMA) markers. While some spheroids with lumen were present when SCp2 and SCg6 cells were co-cultured in 3D, most structures were clusters of cells lacking a lumen (Supplementary Figure 2F). Importantly, none of these structures were bilayered. However, in MCF-12A and Hs 578Bst co-cultures, bilayered acini were produced (Figure 6). These acini were composed of an inner layer of luminal cells surrounded by a discontinuous basket-like network of myoepithelial-like cells (Figure 6A & B). Moreover, a lumen was present in the center of the acini (Figure 6A & C). These results demonstrate that it is possible to create a 3D model of a human bilayered acini *in vitro*, using cell lines.

## Discussion

To fully understand the mechanisms that lead to breast cancer, we first need to understand how a healthy mammary gland functions. Developing a surrogate model of the normal human breast that mimics the architecture and function of the actual organ will help increase our understanding of how breast tissue develops and how specific deregulations, of intercellular junctional complexes for example, influence carcinogenesis. Here we report the production of a relevant 3D heterotypic co-culture model of the functional unit of the mammary gland, the bilayered acinus, consisting of two different cell lines, human myoepithelial-like Hs 578Bst and luminal MCF-12A cells. These spherical bilayered structures consisted of a lumen surrounded by an inner layer of luminal cells enveloped by a basket-like network of myoepithelial cells, similar to what is typically observed *in vivo*.

### The important role of myoepithelial cells

For many years, myoepithelial cells were mostly ignored in mammary gland studies, as it was thought that their role was limited to contraction-inducing transportation and ejection of milk during lactation. We now know that myoepithelial cells are crucial for the proper differentiation and function of the epithelium. Among their functions, they allow paracrine regulation and crosstalk within the epithelium while playing an active role in tissue remodeling and polarization of luminal cells. Such functions are critically dependent on bidirectional communication between myoepithelial and luminal cells [3–5,13]. Moreover, a growing body of evidence demonstrates that the tumor microenvironment plays a critical role in cancer progression [24]. Whether cancer cells induce remodeling of the architecture and/or changes in tissue architecture promote cell tumorigenicity is unclear. It is likely that gene expression is at least in part dictated by the interactions between epithelial cells and the stromal elements, including stromal cells, proteins of the ECM and other soluble factors [24]. As a result, ECM is considered as an active participant in cellular differentiation, rather than a passive bystander. Conversely, cells contribute to the formation of the epithelial microenvironment by producing components of the basement membrane such as collagen, laminins and fibronectin [3]. Because they lie on the epithelial side of the basement membrane, myoepithelial cells are uniquely positioned to accomplish most of the interactions between the epithelium, the basement membrane and the ECM. Myoepithelial cells are crucial mediators of ductal elongation and invasion within the stroma, as they secrete proteins and molecules required for the remodeling of the ECM such as maspin, amyloid β-protein precursor/protease nexin-II and matrix metalloproteinases [3–5]. As a result, dysregulation of myoepithelial cells functions has been associated with the loss of a polarized epithelium, developmental defects and tumorigenesis [6–9]. Accordingly, myoepithelial cells are often considered natural tumor suppressors due to their ability to build a physical and chemical barrier against uncontrolled growth, tumor cell invasion and angiogenesis [42,43]. Therefore, to fully understand both the normal development of mammary gland and breast tumor progression, as well as to study more specifically the direct relationship between luminal and myoepithelial cells of the acinus [12], heterotypic models in which human mammary myoepithelial cells are introduced in a human mammary luminal cell culture must be developed.

### Human luminal & myoepithelial-like commercial cell lines can form bilayered acini, similar to complex 3D structures from primary cultures

Many techniques have been explored to model and study the human mammary gland *in vitro*. Malignant as well as nonmalignant mammary cells have traditionally been studied as monolayers on plastic cell culture dishes, thereby losing their morphological organization and tissue-specific function [16]. Fortunately, progress in tissue engineering and biomaterials have provided researchers with innovative techniques that are now allowing to explore the possibilities of 3D culture, thus bridging the gap between *in vitro* monolayer cell culture models and expensive *in vivo* whole-animal systems [40]. 3D culture systems promote expression of tissue-specific functions and cellular processes by allowing cells to self-assemble and to receive cues from their neighboring cells and the surrounding ECM, which cannot be achieved when cells are plated on plastic cell culture dishes in 2D [40,44]. 3D models are particularly useful for the study of protein and gene functions, along with signaling pathways in a physiologically relevant context [44].

There has been a wide range of 3D culture models, using Matrigel-based matrices, developed in an attempt to replicate the epithelium of the human mammary gland *in vitro*. For instance models using nontumorigenic human mammary luminal cells lines, such as MCF-10A [34], HMT 3522 S1 [36,45] or MCF-12A [35] have been reported. When grown in Matrigel, all these cells lines were able to form organized spheroids with a central lumen, similar to breast acini morphology. In opposite, tumorigenic human mammary luminal cells lines form disorganized, proliferative and nonpolar colonies [33,36]. While these models brought important insights on the structure and the polarization of acini, and the lack of defined structures for breast cancer cells, these models fail to consider the crucial role of myoepithelial cells in the formation of a polarized epithelium *in vivo*.

A few studies have been published with 3D models composed of more than one cell type. For instance, human luminal MCF-10A cells were co-cultured with primary culture of human mammary fibroblasts [46]. In another study, primary cultures of human luminal and myoepithelial cells were co-cultured [47]. In an even more complex model, human luminal MCF-10A cells were co-cultured with primary cultures of human mammary fibroblasts and adipose-derived stem cells [38]. Cells in these co-culture models displayed more differentiated morphological phenotypes and functional activity than in less complex monocultures [38]. Likewise, these co-cultures facilitated the study of cellular crosstalk in the breast [47]. Notably, most of these studies used primary cultures of breast cells. It is believed that primary cells in a 3D mammary gland model enable more physiologically relevant studies such as lineage commitment and plasticity [48], and that normal *in vivo* signaling pathways is more preserved compared with immortalized cell lines [49]. However, some of the downsides of these models are the increased complexity of working with primary cells, the heterogeneity of the samples and the difficulties in genetically manipulating the cells. While this heterogeneity is suitable to study tumor cell biology as it reproduces more closely the interpatient and intratumor variability, it renders mechanistic studies on the role of particular protein during mammary gland development more difficult. On the opposite, commercially available cell lines represent a more homogeneous population that can easily be genetically modified to isolate the role of particular proteins or pathways [38,50]. As such, our model using cell lines represents a more reproducible and manipulable model to study the role of the bidirectional crosstalk between luminal and myoepithelial cells within the mammary epithelium. A similar model using luminal and myoepithelial cell lines combined with primary fibroblast was developed few years ago to study breast cancer, but using cells that are not commercially available, thus limiting its use [39]. Because MCF-12A and Hs 578Bst cell lines are commercially available, the scientific community will therefore be able to use and even improve this model.

### Limitations of our model

Although we successfully obtained bilayered acini, about 50% of the acini formed when co-cultured luminal and myoepithelial cell were bilayered, while the other 50% was formed of luminal cells only. A possible explanation lies in the use of Matrigel. Indeed, it has been reported that co-culturing isolated myoepithelial and luminal cells in type I collagen-based matrix promoted their rearrangement into bilayered acini, while Matrigel did not promote such rearrangements [10,47]. Matrigel is a laminin-rich basement membrane extract. It has been demonstrated that laminin is required for adequate luminal cell polarization in the mammary gland [10], and that myoepithelial cells produce an important amount of this laminin *in vivo* [51]. Consequently, we speculate that in a Matrigel laminin-rich matrix, as laminin is already present, there might be no incentive for luminal cells to coalesce with myoepithelial cells in bilayered structures. On the other hand, in a type I collagen-based matrix in which no laminin is present, the luminal cells might coalesce as they require the laminin produced by myoepithelial cells to promote their assembly into co-units. Yet we still managed to produce bilayered acini of luminal and myoepithelial cell lines embedded in a Matrigel matrix in our model. Other matrices, such as artificial scaffold or type I collagen-based matrix, might help increasing the ratio of bilayered versus luminal cells-only acini. Increasing the myoepithelial to luminal cell ratio in co-culture might also favor the formation of bilayered acini.

The types of cell lines used also seem to a have limitations. In the experiment herein, murine luminal cells SCp2 and myoepithelial cells SCg6 did not interact to form bilayered acini when co-cultured in Matrigel. Both cell lines either formed monolayer acini or irregular structures, but did not coalesce (Supplementary Figure 2). This might be explained by the fact that SCp2 and SCg6 cells have less differentiated phenotypes. In fact, western blot analysis showed that both SCp2 and SCg6 expressed luminal marker E-cadherin and myoepithelial markers K14 and muscle-specific caldesmon-1 (Supplementary Figure 1). Moreover, they showed a high plasticity when cultured in 2D, suggesting stem-like properties (data not shown). Finally, while MCF-12A and Hs 578Bst cells have more differentiated phenotypes and are considered as nontumorigenic, they remained immortal cells. Therefore, signaling and protein expression is surely not exactly reproducing those of *in vivo* luminal and myoepithelial cells. This is supported by the fact that Hs 578Bst cells express p63, α-SMA, calponin-1 and caldesmon-1, markers that are specific for breast myoepithelial cells [52,53], but not K14. Nevertheless, the fact that they form bilayered acini *in vitro* represents an improvement toward more complex, manipulable, reproductive and representative *in vitro* models to mimic the bilayered mammary gland epithelium and study the role of bidirectional communication between the two layers of epithelial cells.

## Conclusion

To the best of our knowledge, this study is the first to use two types of human mammary cell lines cultured together in Matrigel to form bilayered acini, offering significant advantages over previously described models that used monocultures of cell lines or co-culture of primary cells. The critical advantage of our model remains the use of commercially available cell lines that ensure a manipulable, reproducible and physiologically relevant human mammary gland model. Our model will allow the study of the critical role of myoepithelial cells, and of the interactions between myoepithelial and luminal cells in mammary gland development and during breast cancer progression.

## Future perspective

Although we successfully obtained bilayered acini when we co-cultured luminal and myoepithelial human cells, about 50% of the acini formed were bilayered while the other 50% was formed of luminal cells only. Future experiments will be designed using alternative matrices or scaffolds to determine whether they will allow luminal cells to coalesce in bilayered structures with myoepithelial cells in a better proportion. In addition, to further characterize the bilayered acini, we intend to use other markers of luminal and myoepithelial cells. These differentiation markers include K18 and MUC1, as well as calponin, p63 and caldesmon-1, for luminal and myoepithelial cells, respectively.

Studies have demonstrated that luminal cells isolated from human or mice tissue, or even some cell lines, can produce milk when cultured in 3D [54–56]. This is thought to be linked to increased differentiation of luminal cells [56]. To the best of our knowledge, MCF-12A must be exposed to lactogenic hormones (i.e., prolactin) to express appreciable levels of β-casein or whey acidic protein (WAP). Those hormones were not added to our culture, and accordingly, we were not able to detect β-casein or WAP by western blot in MCF-12A cells (data not shown). However, we are planning to determine whether 3D cultures, with or without lactogenic hormones, and with or without being co-cultured with Hs 578Bst cells would induce/increase milk protein expression in MCF-12A cells in future experiments.

Finally, our main goal is to understand how bidirectional crosstalk, particularly cell–cell interactions between luminal and myoepithelial cells, relates to acini polarity, to luminal cell differentiation and to the ‘natural tumor suppressor’ role of myoepithelial cells. To investigate these questions, key junctional proteins, such as connexins, cadherins and catenins, will be inhibited by Clustered Regularly Interspaced Short Palindromic Repeats (CRISPR) technology or siRNA in luminal cells or in myoepithelial cells only, or in both layers, and the effects on double-layered acini formation will be analyzed. This will allow for a better understanding of the role of the bidirectional crosstalk between the two layers of cells, and how it influences the epithelium differentiation and function.

**Executive summary**

- The whole ramified lobulo-alveolar system of the breast epithelium consists of an inner layer of luminal cells bordering a central lumen and surrounded by an outer layer, mainly comprised of myoepithelial cells.
- The breast development and function are critically dependent on bidirectional communication between myoepithelial and luminal cells.
- The stroma directly surrounding the epithelium guides the development of the epithelium and the epithelium reciprocally influences its microenvironment.
- Cells cultured in 3D in extracellular matrix can better form cell–cell and cell–extracellular matrix interactions, establish appropriate cell-signaling pathways to maintain tissue function and mimic the cellular processes occurring in the human body.
- Most 3D cell culture systems mimicking mammary gland acini are either monocultures, using only the luminal cells, or co-cultures composed of primary cells isolated from human tissues and difficult to genetically manipulate; these models are thus not appropriate to study mechanisms involved in the luminal–myoepithelial cells crosstalk.
- To fully understand both the normal development of mammary gland and the progression of breast tumor, as well as to study more specifically the direct relationship between luminal and myoepithelial cells of the acinus, heterotypic models in which human mammary myoepithelial cells are introduced in a human mammary luminal cell culture must be developed.
- This study aimed to develop a model representing the breast bilayered acini that can be genetically manipulated and easily reproduced by using cell lines; such a model will be accessible to the scientific community and allow more mechanistic studies in the understanding of the biology of the bilayered acini.
- MCF-12A and Hs 578Bst human cells showed more differentiated phenotypes than SCp2 and SCg6 mouse cells.
- MCF-12A and SCp2 luminal cells, as well as SCg6 myoepithelial cells, form spheroid structures when embedded in Matrigel, while Hs 578Bst do not.
- Luminal MCF-12A and myoepithelial-like Hs 578Bst commercial cell lines can form bilayered acini, similar to complex 3D structures from primary cultures.
- This model represents a first step toward more complex, manipulable, reproductive and representative *in vitro* models to mimic the bilayered mammary gland epithelium and study the role of bidirectional communication between the two layers of epithelial cells.
