# Supplementary material for: Luminal MCF-12A & myoepithelial-like Hs 578Bst cells form bilayered acini similar to human breast
Source: Future Sci OA. 2018 Jun 28;4(7):FSO315. doi: 10.4155/fsoa-2018-0010 (PMC6088263; doi:10.4155/fsoa-2018-0010)
Supplement: Supplementary file 1 [file fsoa-04-314-s1.pdf]

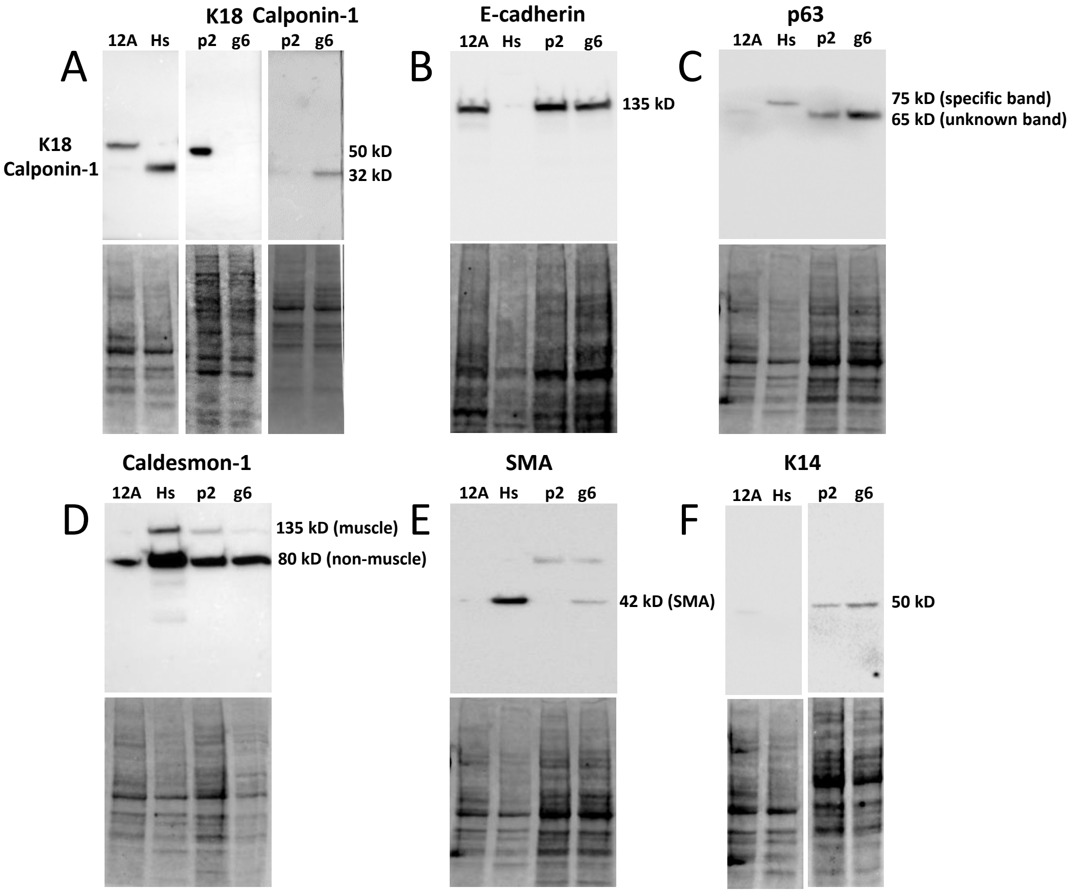

Supplementary figure 1. Human luminal epithelial MCF-12A and myoepithelial-like Hs 578Bst cells have more differentiated phenotypes than murine luminal epithelial SCp2 and myoepithelial SCg6 cells. MCF-12A, Hs 578Bst, SCp2 and SCg6 cells were cultured in 2D. Western blot analysis were performed using antibodies against (**A**) cytokeratin 18 (K-18) and (**B**) E-cadherin luminal markers, as well as (**A**) calponin-1, (**C**) p63, (**D**) caldesmon-1, (**E**) SMA and (**F**) Cytokeratin 14 (K14) myoepithelial markers, respectively. Note that blot A, left panel, represents the same membrane sequentially probed with K18 and calponin-1. Similarly, blots C and D represent the same membrane sequentially probed with p63 and SMA. For all blots, bottom panels show total lane proteins.
