# Supplementary material for: Luminal MCF-12A & myoepithelial-like Hs 578Bst cells form bilayered acini similar to human breast
Source: Future Sci OA. 2018 Jun 28;4(7):FSO315. doi: 10.4155/fsoa-2018-0010 (PMC6088263; doi:10.4155/fsoa-2018-0010)
Supplement: Supplementary file 2 [file fsoa-04-314-s2.pdf]

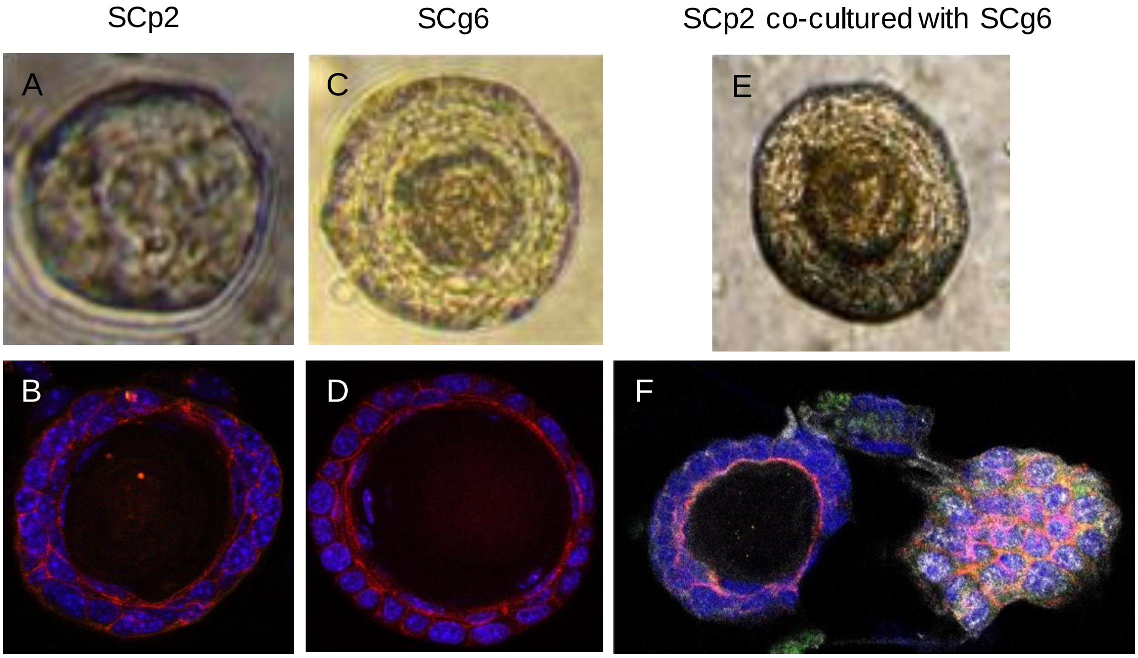

Figure supplementary 2. Co-cultures SCp2 and SCg6 cells did not form bilayered acini in Matrigel. (**A, C, E**) Representative single optical microscopy images of SCp2 cells (**A**), SCg6 cells (**B**) and SCp2 cells co-cultured with SCg6 cells (**E**) embedded in Matrigel. (**B, D, F**) Cells were immunolabeled with E-cadherin (red) and SMA (white). Nuclei are stained with DAPI (blue). SCg6 cells formed spheroids structures with lumen (**C, D**), similar to SCp2 (**A, B**). When SCp2 were co-cultured with SCg6 (**E, F**), some spheroids had a lumen while other did not. SMA was present only on structures without lumen (**F**). SCp2 and SCg6 cells did not form bilayered spheroid with a lumen in co-culture.
